# Use of Self-Assembling Peptides to Enhance Stem Cell Function for Therapeutic Angiogenesis

**DOI:** 10.1155/2018/4162075

**Published:** 2018-06-13

**Authors:** Hyung Sub Park, Geum Hee Choi, Daehwan Kim, Tae Woo Jung, In Mok Jung, Jung Kee Chung, Taeseung Lee

**Affiliations:** ^1^Department of Surgery, Seoul National University College of Medicine, Seoul, Republic of Korea; ^2^Department of Surgery, Seoul National University Bundang Hospital, Gyeonggi, Republic of Korea; ^3^Department of Surgery, Seoul Metropolitan Government Seoul National University Boramae Medical Center, Seoul, Republic of Korea

## Abstract

The use of nanomaterials for biomedical applications has become a promising field in regenerative medicine. Self-assembling peptides (SAPs) have been proposed as a good candidate because they are able to self-assemble into stable hydrogels and interact with cells or molecules when combined together. This in turn can lead to the improved survival or action of cells or molecules to obtain the desired effects. In this study, we investigated whether the combination of mesenchymal stem cells (MSCs) with SAPs could improve angiogenesis in ischemic hindlimbs of rats compared to MSC or SAP treatment alone. The combination of MSCs and SAPs showed an overall higher expression of angiogenesis markers on fluorescent immunohistochemical analysis and a lower degree of fibrosis and cell apoptosis, which in turn led to an overall tendency for improved perfusion of the ischemic hindlimbs. Finally, SAPs also showed the ability to recruit endogenous host MSCs into the site of action, especially when modified to incorporate substance P as a functional motif, which when injected with exogenous MSCs, allowed for the dual presence of MSCs at the site of action. Overall, these results suggest that SAPs can be applied with stem cells to potentiate angiogenesis, with potential therapeutic application in vascular diseases.

## 1. Introduction

Stem cell therapy for treatment of vascular diseases is a promising field of research. This is especially so for cases where ischemic insults and consequent end-organ perfusion defects cannot be overcome. In the clinical setting, peripheral vascular diseases such as Buerger's disease or ischemic diabetic foot are related with poor wound healing outcomes and high amputation rates mainly due to the failed resolution of the underlying ischemia using currently available revascularization methods. In such cases, an increase in angiogenesis can overcome the situation by the formation and outgrowth of new vessels. The role of stem cells in therapeutic angiogenesis has been widely investigated, and its possible application in multiple vascular diseases has also been studied previously [1, 2]. However, the problem with stem cell therapy lies in its limited survival rate and durable effects, especially when administered by external injection [3]. Stem cells can be administered locally or systematically, and depending on the mode of administration, the target for stem cell potentiation may vary. In the case of systemic delivery, an improvement in stem cell homing can be a target for enhanced efficacy, while for local delivery, enhancing stem cell survival can be more important. In any case, new technologies are being studied to increase survival and prolong the effects of exogenously administered stem cells. One of these include biological scaffolds, which can provide a 3-dimensional microenvironment in the form of extracellular matrices (ECMs) to protect the stem cells from apoptosis while prolonging their effects *in vivo*.

Self-assembling peptides (SAPs) are one form of biological scaffold which typically consist of 8–16 amino acids with alternating hydrophilic and hydrophobic residues that assemble itself into a 3-dimensional configuration when administered into the site of action [4]. The hydrophilic portion consists of alternatively repeating units of positively and negatively charged amino acids which form stable *β*-sheets in water, which in turn combine to form flexible nanofiber hydrogels of 7–20 nm in diameter under physiologic conditions [5, 6]. In this way, these hydrogels are able to support 3-dimensional culture of not only different cell types, but also smaller proteins such as growth factors or chemokines through noncovalent bonding, allowing for controlled delivery and sustained release. These SAPs have the advantage of being versatile, easy to manufacture, cheap, biocompatible, noncytotoxic, and biodegradable, making them an attractive source for application in regenerative medicine [7]. SAPs can also be modified in a wide variety of ways to provide unique features and perform specific functions, allowing for promising applications in the field of drug delivery, tissue engineering, and biosensor device applications [7–9]. SAPs are known to have an ECM-resembling configuration for cell and protein delivery, but they have also been shown to have intrinsic mesenchymal stem cell- (MSC-) recruiting ability, thus being able to recruit circulating host MSCs into the site of action. One of these SAPs is known as RADA (16-I or 16-II), which has been demonstrated in previous animal studies to have the ability to recruit host MSCs into ischemic limbs to improve hindlimb perfusion [10]. Its potential for recruitment can be further enhanced if modified to combine a functional motif in the sequence. Despite the numerous advantages and potential applications of SAPs, there are also disadvantages, mainly related to limited availability of data. The kinetics and reproducibility of protein or cell delivery *in vivo* has not been evaluated yet, and the extent of degradation is not well known either. Further immunological testing is also needed.

In this study, we examined whether the combined administration of MSCs with SAPs into ischemic hindlimbs of rats could increase angiogenesis and decrease cell apoptosis and fibrosis, probably by mechanisms related to an increase in MSC survival, which in turn could lead to improvements in perfusion. The role of SAPs in the recruitment of circulating host MSCs was also investigated, and the possibility of a synergistic effect between exogenously administered MSCs and endogenously recruited host MSCs was verified.

## 2. Materials and Methods

### 2.1. Isolation and Culture of Murine MSCs

MSCs were isolated from the femoral bones of 3-4 wk-old Sprague-Dawley male rats using the Ficoll-Hypaque medium (1.077 g/mL, GE Healthcare Life Sciences) and cultured in T-25 culture dishes using Dulbecco's Modified Eagle's Medium (DMEM, Sigma-Aldrich), 10% fetal bovine serum (FBS, Gibco BRL), 100 U/mL penicillin, 100 *μ*g/mL streptomycin (Gibco BRL), and 2 mM L-glutamate (Gibco BRL) at 37°C and 5% CO_2_ atmosphere. After 3–5 passages, cells were sorted using the MACS and CD105 MultiSort Kit (PE, Miltenyi Biotec Inc.). These cells were then characterized for CD105, CD73, CD29, and CD45 (known markers for rat bone marrow MSCs in particular) [11, 12] with either mouse immunoglobulin-FITC or mouse immunoglobulin-PE using a BD FACSCalibur™ Flow Cytometer (BD Biosciences). These cells were then transfected with a lentivirus (Macrogen) tagged with a green fluorescent protein (GFP) marker for use in *in vitro* and *in vivo* studies.

### 2.2. Self-Assembling Peptide Preparation

The self-assembling peptide used in this study was RADA 16-II (Peptron) having the configuration AcN-RARADADARARADADA-CONH_2_ (hereinafter simplified as RADA), with its characteristics having been already described elsewhere [13]. RADA was dissolved in sterile 295 nM sucrose at 1% (wt/vol) and sonicated for 30 min.

### 2.3. *In Vitro* Culture and MTT Assay of MSCs and SAPs

For colony-forming unit fibroblast (CFU-F) identification, DMEM solution and 1.2% agar were mixed and placed on each well in a 6-well plate. Once solidified, MSCs (2500 cells) or MSCs + RADA were mixed with 0.6% agar, dispensed on different wells, and cultured at 37°C and 5% CO_2_ atmosphere. After 14 days of culture, the wells were stained with crystal violet for 1 hr and observed under a microscope system (Olympus). Colonies were counted at 100x magnification in 10 different random points.

For the MTT assay, a 96-well plate was used where MSCs (1 × 10^4^ cells) or MSCs + RADA were placed on different wells with culture medium and cultured at 37°C and 5% CO_2_ atmosphere. A control group was also used where only culture medium was placed. Then, 10 *μ*L of the MTT solution (Cell Counting Kit-8, Dojindo Molecular Technologies, Inc.) was added into each well and placed in a CO_2_ incubator where an ELISA reader (SpectraMax Plus 384, Molecular Devices) was used to measure the absorbance at 450 nm wavelength at 0.5, 1, 1.5, and 2 hr.

For live cell screening analysis, 1 × 10^4^ cells of MSCs (MSC group) or MSCs with RADA (MSC + RADA group) were placed in a 6-well plate and monitored using the IncuCyte Live Cell Analysis System (Essen BioScience) at 37°C and 5% CO_2_ atmosphere. The culture media was changed at 3 days and the screening of cell growth was recorded by the IncuCyte system software for 7 days.

### 2.4. Animal Preparation

Hindlimb ischemia models were created in 3-4 wk-old Sprague-Dawley rats by applying continuous isoflurane general anesthesia. A longitudinal incision was made in the left hindlimb at the thigh level, and the femoral artery was identified. The femoral artery was meticulously dissected along its entire course and was removed after ligation of the proximal and distal ends. Care was taken not to injure the surrounding structures including the femoral vein and nerve. A total of 32 rats were divided into 4 groups: MSC, RADA, combination of MSCs + RADA, and control. For the MSC group, 1 × 10^7^ cells were injected intramuscularly at 4-5 different locations in the thigh immediately after femoral artery excision and at postoperative day 3. In the same manner, RADA was mixed in 200 *μ*L of phosphate-buffered saline (PBS) and injected for the RADA group, and the combination group included a mixture of 1 × 10^7^ MSCs and RADA in 200 *μ*L of PBS, while for the control group, the same amount of saline was injected. This study was approved by the Institutional Animal Care and Use Committee (IACUC) of Seoul National University Bundang Hospital.

### 2.5. Measurement of Hindlimb Perfusion with Laser Doppler

Measurements of perfusion were carried out using a laser Doppler perfusion monitoring system (Periflux System 5000, Perimed AB) immediately after the surgery and at days 7, 14, and 28. A standardized protocol was used for the measurement of perfusion in an attempt to decrease the variability inherent to this method, where the anesthetized rats were placed in the same position with the leg fixed in a slightly extended position and under a warm environment using a heating system. The probe was then placed at the same place every time on the forefoot and fixed with adhesive tapes against the table. The probe was set at 37°C and the measurement was carried out for 2 min once the set temperature was achieved. The mean perfusion was obtained as perfusion units (PU) for each rat at different time points for the different groups.

### 2.6. Immunohistochemical (IHC) Staining and Reverse Transcriptase Polymerase Chain Reaction (RT-PCR)

The study rats were harvested at 7, 14, and 28 days after the measurement of perfusion by removing their thigh muscles and were fixed in 10% formalin for 24 hr. The tissues were then embedded in paraffin and sliced at 5–7 *μ*m thickness. Immunohistochemical analysis was performed for MSC markers CD105, CD90, and CD29 by using their respective antibodies: anti-CD105/Endoglin antibody (Abcam), anti-CD90/Thy1 antibody (Abcam), and anti-CD29/integrin beta-1 antibody (Abcam). For measurement of angiogenesis, IHC staining was performed for angiogenesis markers using primary polyclonal antibodies von Willebrand factor (vWF 1 : 100, Dako), CD31 (1 : 200, Abcam), and *α*-smooth muscle actin (*α*-SMA 1 : 200, Abcam) at 4°C overnight and then with goat anti-rabbit IgG (rhodamine, Abcam) as secondary antibody at 4°C for 3 hr. Nuclei counterstaining was performed with 4′,6-diamidino-2-phenylindole (DAPI, Vector Laboratories). Capillary density was measured by counting the positive stains at 6 different random fields for 3 times under 100x magnification microscopy.

For RT-PCR analysis, harvested tissues were preserved at 4°C in RNAlater (Qiagen) and the total RNA was extracted using an RNeasy Mini Kit (Qiagen) according to the manufacturer's instructions. Quantification of the extracted RNA was done at 260 and 280 nm wavelengths using a NanoDrop Spectrophotometer (Thermo Fisher Scientific). The forward and reverse primers used for CD31, *α*-SMA, vWF, and GAPDH (control) were as follows: CD31 forward 5′-GTGGAACTGGGGACAAAGAA-3′, reverse 5′-TCCGGATGAATTCTGAGGTC-3′; *α*-SMA forward 5′-TGCTGTCCCTCTAGCCTCT-3′, reverse 5′-GAAGGAATAGCCACGCTCAG-3′; vWF forward 5′-TGCTGCCCAGAGTATGAGTG-3′, reverse 5′-GATGTTCCTCTGCTGCACAA-3′; GAPDH forward 5′-TGCCACTCAGAAGACTGTGG-3′, reverse 5′-GCATGTCAGATCCACAATGG-3′. PCR was performed using a PTC-200 PCR Thermal Cycler (MJ Research) under the following cycling conditions: 94°C for 2 min (initial step), 94°C for 30 s (denaturation), 53–55°C for 45 s (annealing), and 72°C for 30 s (elongation) for a total of 35 cycles; 72°C for 10 min was used for the final extension. Electrophoresis of PCR products in 1-2% agarose gel with ethidium bromide was performed and images were analyzed using a bioimage analyzer EXT-20MX (Vilber Lourmat). Band intensities were normalized against GAPDH.

### 2.7. Apoptosis and Fibrosis

Apoptosis at the cellular level was measured using the terminal deoxynucleotidyl transferase deoxyuridine triphosphate nick end labeling (TUNEL) method. A TUNEL assay kit (Millipore) was used for staining of harvested tissues at days 7, 14, and 28, and the apoptosis ratio was calculated by counting the number of TUNEL-positive nuclei and dividing it by the total number of nuclei at 5 different random fields (200x magnification) using a Mantra Quantitative Pathology Imaging System (PerkinElmer).

Degree of fibrosis formation was analyzed by staining the paraffin-embedded sections with Masson's trichrome stain and by calculating the area of fibrosis (per mm^2^) in 10 different random fields (100x magnification) using a Mantra Quantitative Pathology Imaging System (PerkinElmer).

### 2.8. Modification of SAPs for Enhanced Recruitment of Intrinsic MSCs

In order to enhance recruitment of intrinsic MSCs into the site of action, RADA was modified to incorporate a specific motif required to achieve the desired action. For this purpose, substance P was used, which is well known as a neurotransmitter at the peripheral terminals of sensory nerve fibers, but has also been demonstrated to have a role in the mobilization and recruitment of endogenous MSCs into the site of action [14]. The new peptide, named RADA-SP (Peptron), had the configuration AcN-RARADADARARADADAGGRPKPQQFFGLM-CONH_2_. A subgroup analysis was performed where RADA-SP (RSP) was added to MSCs with RADA and injected into ischemic hindlimbs. The other 3 groups consisted of control, MSCs, and RSP, and the hindlimbs were harvested at 7, 14, and 28 days for IHC staining of stem cell markers CD105, CD90, and CD29 (as previously described).

### 2.9. Statistical Analysis

All continuous variables are presented as mean ± standard error of the mean (SEM). Statistical analysis between multiple comparison groups was done by one-way analysis of variance (ANOVA) with post hoc testing using the Tukey method, while 2 group comparisons were performed using the *t*-test. IBM SPSS Statistics 20 (IBM) was used for analysis, and differences were considered statistically significant when *P* < 0.05.

## 3. Results

### 3.1. *In Vitro* Characteristics of MSCs and SAPs

**Figure 1:**
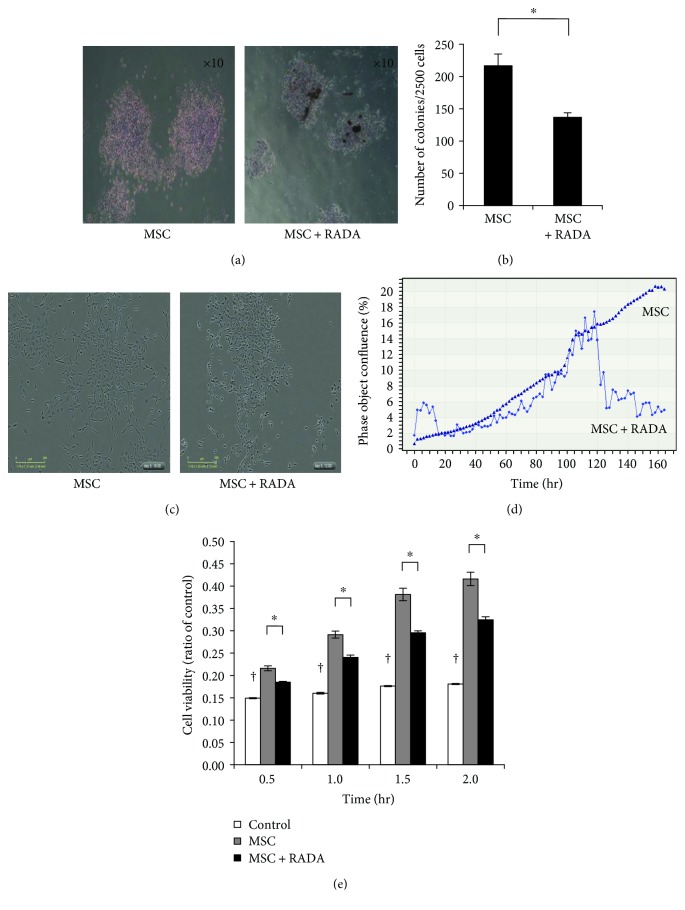
Combination of MSCs with RADA in *in vitro* cultures, demonstrating that their combination showed lower cell viability than MSCs alone. (a) Representative cell culture showing formation of colonies by MSCs and the combination of MSCs and RADA (10x magnification). (b) Quantification of colonies formed by MSCs and MSCs + RADA. The combination group showed significantly lower colony-forming units than MSCs alone. (c) Representative cell culture showing survival of MSCs and the combination of MSCs and RADA after 160 hours of live culture (10x magnification), showing an overall lower number of cells in the combination group compared to MSCs alone. (d) The lower cell growth in the combination group is shown in this quantification of cell growth for MSCs alone and MSCs + RADA, as measured by live cell screening analysis. (e) Quantification of cell viability by MTT assay also demonstrates that MSCs + RADA showed significantly lower cell viability than MSCs alone when compared to control. ^†^*p* < 0.05 for control versus all other groups. ^∗^*p* < 0.05.

Isolated and cultured murine MSCs exhibited characteristic MSC markers (positive CD105, CD29, and CD73, and negative CD45 markers) and showed multilineage differentiation to osteogenic, neurogenic, endothelial, and smooth muscle cell differentiation *in vitro*, while features of cell senescence were not observed after 15 passages (data not shown). When MSCs were cultured *in vitro*, they formed characteristic colonies, but when cocultured with RADA, their colony-forming ability was decreased (Figures 1(a) and 1(b)). Live cell screening analysis also demonstrated that MSCs continued to grow up to 160 hours but the combination of MSCs and RADA showed a decrease in growth after 100–120 hours (Figures 1(c) and 1(d)). The MTT assay also showed that the combination of MSCs and RADA showed significantly lower cell viability compared to MSCs alone (Figure 1(e)). These results demonstrate that, *in vitro*, the combination of MSCs and RADA did not improve cell survival of MSCs.

### 3.2. *In Vivo* Cell Characteristics and Angiogenesis

**Figure 2:**
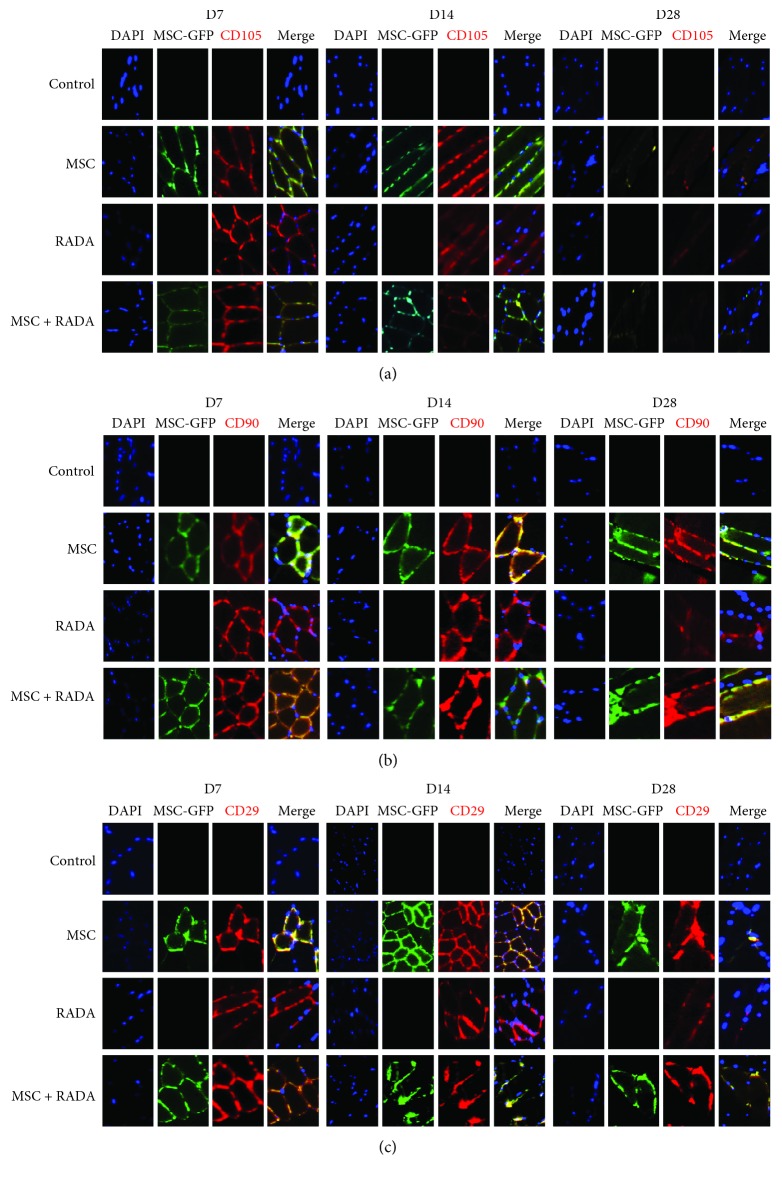
Fluorescent immunohistochemical staining of stem cell markers (a) CD105, (b) CD90, and (c) CD29 for MSCs, RADA, MSCs + RADA, and control (10x magnification). Injected MSCs previously tagged with GFP stained green in the presence of MSCs (second column for each time point). The different stem cell markers were stained for (third column for each time point) and showed the presence of stem cells, not only in the MSC and MSC + RADA group, but also in the RADA-alone group, suggesting the presence of stem cells probably from the recruitment of endogenous MSCs by RADA. The merged images showed a characteristic yellow color from double staining of GFP and stem cell markers (second and fourth row for the different stem cell markers at different time points), yet there was no presence of endogenously recruited MSCs in the combination group which would have shown a red stain only in the merged images (fourth rows).

Harvested tissues at different time points (7, 14, and 28 days) were stained for MSC markers CD105, CD90, and CD29 to detect for the presence of MSCs (Figure 2). The control group did not show any positive stain for all MSC markers, while MSCs showed a positive stain for all three markers at different time points. Since injected MSCs were also tagged with GFP, a positive GFP stain was observed (second column). RADA also demonstrated a positive stain for MSC markers which was not observed in the control group, demonstrating that SAPs have the potential to recruit MSCs to the site of action. For the MSC + RADA combination group, the tissues stained positive for GFP and for MSC markers, but when merged together, only the yellow stain was visible. This meant that only the injected MSCs were present in the tissues and no MSCs were recruited by RADA, which would have stained red after merging (since GFP is not present). In terms of stem cell presence or survival, there was a definite difference between control and the 3 groups, but there was no visible correlation between the 3 groups or between different time points.

**Figure 3:**
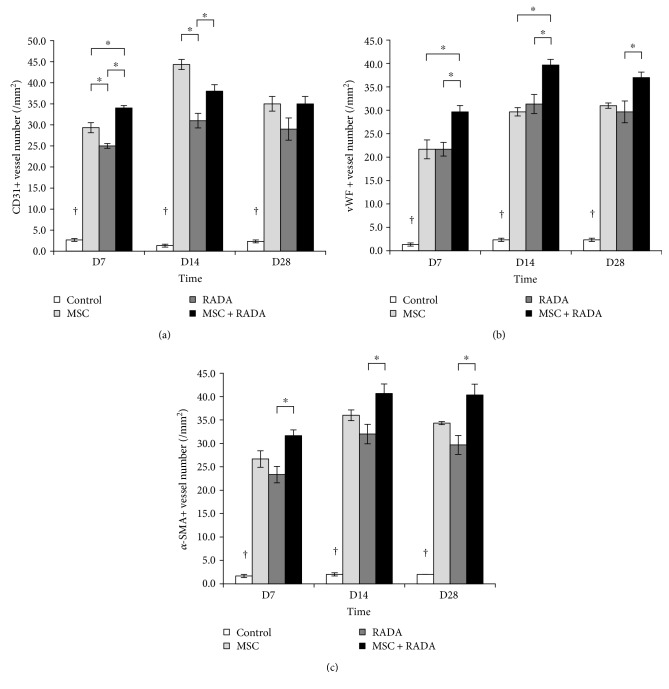
Quantification of angiogenesis from immunohistochemical staining for MSCs, RADA, MSCs + RADA, and control. The markers (a) CD31, (b) vWF, and (c) *α*-SMA were used to determine angiogenesis and the overall results demonstrate an increase in marker expression in the 3 groups with respect to control, while the MSC + RADA combination group tended to show an overall higher expression compared to MSCs or RADA alone. ^†^*p* < 0.05 for control versus all other groups. ^∗^*p* < 0.05.

IHC staining for angiogenesis markers CD31, vWF, and *α*-SMA was performed from harvested tissues at different time points (Figure 3). The results show that compared to control, all the other groups showed a significant increase in angiogenic markers at all time points. MSCs showed an overall higher (or at least similar) angiogenic potential than RADA, demonstrating that exogenously injected MSCs were stronger than MSCs recruited by RADA in forming capillaries in ischemic tissues. Finally, the combination group showed an overall higher angiogenic potential than both MSCs and RADA alone (although statistically not significant at some time points), suggesting that an increase in survival of MSCs when combined with RADA led to a better ability to form capillaries in ischemic tissues.

RT-PCR to determine the induction of angiogenesis at the genetic level was performed for CD31, vWF, and *α*-SMA from harvested tissues, and there was a significantly higher expression of angiogenic genes for all 3 groups compared to control, but when the 3 groups were compared, there was no overall correlation between the groups (data not shown).

### 3.3. Hindlimb Perfusion Measurements

**Figure 4:**
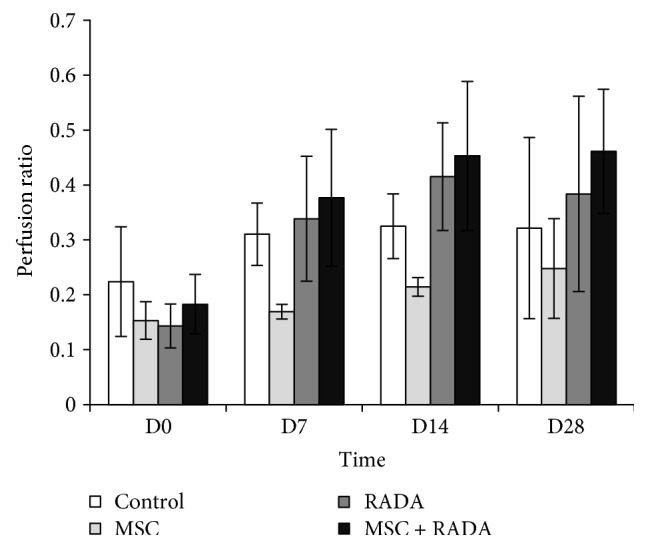
Quantification of hindlimb perfusion by a laser Doppler perfusion measuring system for MSCs, RADA, MSCs + RADA, and control, demonstrating a tendency for better perfusion in the MSC + RADA combination group compared to MSCs or RADA alone, although not statistically significant due to the high variability of the measuring system used.

The perfusion of hindlimbs as measured by laser Doppler was expressed as the ratio of the measured perfusion postoperatively divided by the preoperative baseline perfusion (Figure 4). As expected, at day 0 (immediate postoperation) there was no difference between the 4 groups. Furthermore, there was no statistically significant difference between the 4 groups at the other time points, yet there was tendency for the combination group to show a higher perfusion compared to the other groups at 7, 14, and 28 days.

### 3.4. Apoptosis and Fibrosis

**Figure 5:**
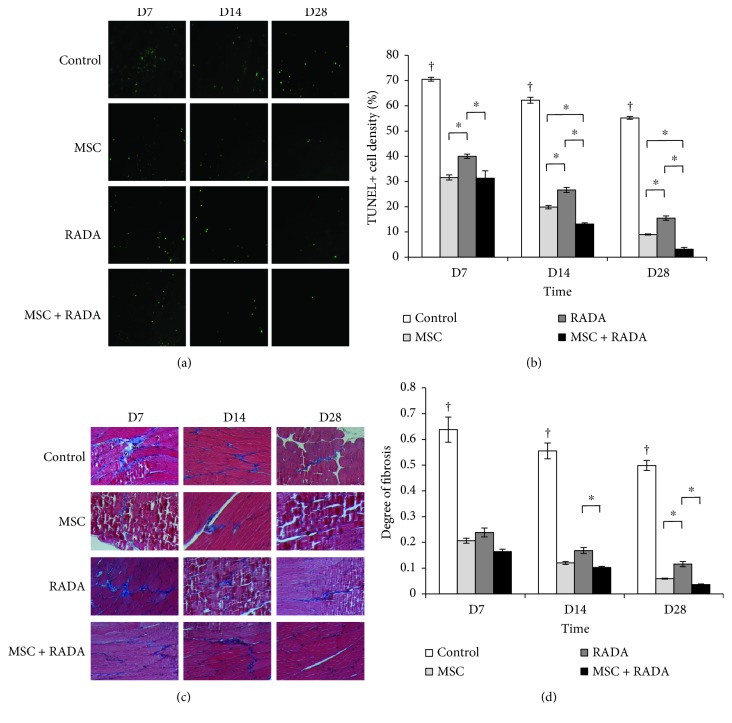
(a) Fluorescent TUNEL staining and (b) quantification of TUNEL-positive stains for the evaluation of apoptosis for MSCs, RADA, MSCs + RADA, and control. There is a statistically significant lower rate of apoptosis in the MSC + RADA group compared to MSCs or RADA alone. (c) Representative Masson's trichrome staining showing fibrosis between muscle fibers (10x magnification) and (d) quantification of degree of fibrosis from immunohistochemical stains for the evaluation of fibrosis for MSCs, RADA, MSCs + RADA, and control. There is an overall tendency for lower fibrosis formation in the MSC + RADA group compared to MSCs or RADA alone. Lower degree of fibrosis and apoptosis correlates with an improvement in limb perfusion from increased angiogenesis. ^†^*p* < 0.05 for control versus all other groups. ^∗^*p* < 0.05.

Apoptosis was determined using TUNEL staining to demonstrate the degree of cell survival from harvested tissues (Figures 5(a) and 5(b)). The control group showed the highest rate of apoptosis compared to all other groups at all time points, with statistical significance. The combination group showed a significantly lower rate of apoptosis, followed by the MSC group and the RADA group. This difference was most definite at 14 and 28 days, suggesting that the combination group had the strongest cell regenerative effect after ischemic injury to the hindlimbs.

The degree of fibrosis formation was also determined from harvested tissues using Masson's trichrome staining (Figures 5(c) and 5(d)). Similar to cell death, the control group showed the highest degree of fibrosis compared to all other groups at 7, 14, and 28 days with statistical significance. Overall, the combination group showed the lowest degree of fibrosis, followed by the MSC group and the RADA group. These differences were mostly significant at 14 and 28 days.

### 3.5. RSP-Mediated Recruitment of Intrinsic MSCs

**Figure 6:**
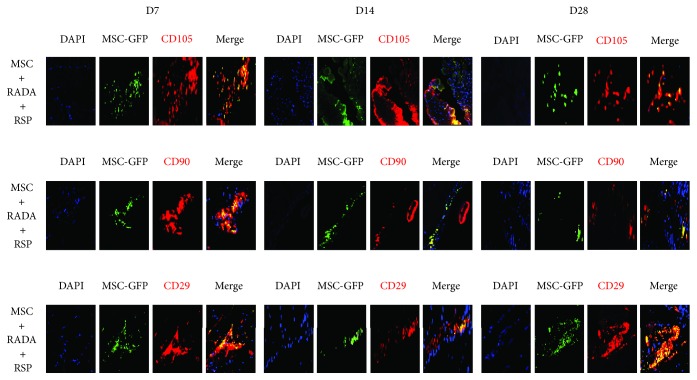
Fluorescent immunohistochemical staining for stem cell markers CD105, CD90, and CD29 from ischemic hindlimbs injected with a combination of MSC, RADA, and RSP (10x magnification). RSP is proposed as being stronger in recruiting endogenous MSCs and therefore the combination of MSCs with RADA and RSP showed the presence of both injected MSCs (yellow stain) and endogenously recruited MSCs (red stain) in the merged images for the different markers at different time points.

A subgroup analysis was performed where RSP was added to the previous combination group and fluorescent IHC was performed for stem cell markers. Similar to the previous results, MSCs and RSP alone showed positive staining for stem cell markers CD105, CD90, and CD29, and injected MSCs also showed positive stain for GFP, thus showing a yellow stain when merged, while the control group did not show any stain for stem cell markers or GFP, as expected (data not shown). In the combined MSC + RADA + RSP group, injected MSCs were stained green on GFP stain, while all MSCs (both injected and intrinsic) were stained red for the respective stem cell markers (CD105, CD90, and CD29). As a result, injected MSCs showed a yellow stain, while endogenously recruited MSCs showed only a red stain on merged images. This was apparent in this new, RSP added, combination group where the merged stains showed both yellow and red stains on the same slide for all stem cell markers at different time points (Figure 6). These results demonstrate that by adding RSP, intrinsic host MSCs were recruited to the site of action in combination with injected MSCs, which was not so apparent when MSCs and RADA were combined alone.

## 4. Discussion

In this study, we evaluated whether the combination of MSCs and SAPs improves angiogenesis in ischemic hindlimbs of rats. SAPs have been demonstrated to provide a niche for improvement in stem cell survival, which in turn leads to prolonged effects. RADA in particular has been demonstrated to improve myocardial infarction when combined with either platelet-derived growth factor-BB (PDGF-BB) [15] or with a combination of PDGF-BB and fibroblast growth factor-2 (FGF-2) [13]. This was achieved by controlled local delivery of the respective growth factors when combined with RADA, while injection of growth factors or RADA alone did not achieve the desired cardioprotective effects. The combination of stem cells with SAPs has also been studied widely in a variety of different animal models, including ischemic hearts [16], osteoarthritis [17], and cartilage defects [18]. In these studies, the role of SAPs was to act as a carrier to increase engraftment of injected stem cells, thereby improving survival and retention of administered cells and thus improving their desired therapeutic effects.

Our study results demonstrated that the combination of MSCs and RADA *in vitro* did not cause any improvement in cell survival. In fact there was a statistically significant decrease in colony formation and cell viability when MSCs were combined with RADA compared to MSCs alone. Live cell screening analysis also demonstrated that after a certain point in cell growth, the combination group showed a sudden decrease in cell proliferation. Similar results were observed in a study by Liu et al. where *in vitro* 3-dimensional culture of human adipose stem cells with RADA 16-I showed a lower proliferation than 2-dimensional culture of adipose stem cells alone, and only when combined with a functionally modified RADA 16-I to include stem cell homing motifs was cell proliferation significantly improved [19]. Stevenson et al. also showed that the incorporation of specific sequences as binding sites into SAPs was associated with increased microvascular network formation, while SAPs without these binding sites showed no network formation [20]. The reason for the lower proliferation of MSCs with RADA in our *in vitro* results is not well understood, but the sudden decrease in cell proliferation after a certain point in time suggests that there may be competitive inhibition of stem cell growth, possibly due to overcrowding. Another possible explanation is that despite the stable formation of a hydrogel network under a physiologic aqueous environment, the self-assembly process of SAPs is known to be affected or even reversed depending on external environmental factors such as pH, temperature, ionic strength, and mechanical forces [21]. It may be possible that the physiologic environment required for stable hydrogel formation and proliferation of stem cells in a 3-dimensional manner may not have been achieved in our *in vitro* cultures.

When MSCs were injected into *in vivo* ischemic hindlimb animal models in combination with RADA, the results were more promising. Fluorescent IHC staining for stem cell markers (CD105, CD90, and CD29) demonstrated the presence of stem cell survival in the different groups at 7, 14, and 28 days. The presence of stem cell markers in the RADA group but not in the control group also demonstrates that RADA has the potential to recruit stem cells, most probably endogenous host stem cells through homing mechanisms into the site of action. In the combination group however, there was the presence of injected MSCs only, which appeared as a yellow stain in the merged image (from the double staining of GFP and stem cell markers), and no presence of endogenously recruited MSCs, which would have stained red in the merged image. Only when RSP, which incorporates a functional motif to achieve a prolonged release of substance P, was added to the combination group could the presence of both injected MSCs and recruited host MSCs be visualized. The reason for the absence of endogenous MSCs in the first combination group (MSCs + RADA) is not well understood, but it can be postulated that by combined injection, the MSCs immediately adhere to the assembled hydrogels, thereby attenuating the recruiting potential of RADA. Another possibility is that the recruiting ability of RADA itself is weak, and therefore cannot overcome the competition against already injected MSCs at the site of action. This is in accordance with a previous study where despite the ability of RADA to recruit endogenous MSCs, the *in vivo* effects were enhanced only when a second RADA with substance P bound as a functional motif into the sequence was added [10]. SAPs are known to be modifiable to perform specific bioactive functions when functional peptide motifs are incorporated into the C termini of the peptide sequence separated by a GG spacer between the motif and the ionic-complimentary sequence [22]. Substance P, in particular, has been shown to enhance the recruitment of endogenous stem cells into implanted scaffolds to increase angiogenesis [23], mainly from circulating cells inside the blood circulation around the local site of action [24]. The addition of RSP to the combination group may have provided a stronger drive for recruitment of circulating host MSCs, which in turn may improve angiogenesis further, although further studies are needed.

The combination of MSCs and RADA resulted in an overall increase in angiogenesis, as shown by the increase in angiogenesis markers CD31, vWF, and *α*-SMA. As stated previously, RADA may have potentiated the effects of MSCs by providing a stem cell niche in the form of a 3-dimensional microenvironment in which MSCs can reside more stably by allowing for cell attachment, migration, and differentiation. In this microenvironment, stem cells were able to increase their angiogenic effect under the ischemic conditions in which they were exposed to by differentiating into endothelial and vascular smooth muscle lineage cells required for new vessel formation. Whether the survival rate of MSCs was actually increased *in vivo* by the addition of RADA was not quantified in this study. We found that their angiogenic effects were improved up to a certain point and the tendency was clear although statistical significance was not achieved at all time points. The lack of statistical significance at 28 days is a limitation, since the improvement in the durability of effects by the combination of MSCs with RADA was a desired effect, yet it may also be possible that by 28 days, the ischemic insult caused by the ligation of the femoral artery may have been restored up to a certain point that the drive for angiogenesis may have attenuated. Although we did not quantify stem cell survival *in vivo*, may reports have already shown that stem cell survival may not be a crucial issue for prolonged effects, since one of the most widely accepted mechanism of action of stem cells is through paracrine effects, releasing potent trophic factors that can modulate the molecular composition of the environment to evoke responses from resident cells [25, 26].

Hindlimb necrosis or color changes were not visible in our experiment, and therefore the improvement in ischemia could not be grossly detected. The use of laser Doppler to verify the improvement in perfusion as a result of angiogenesis showed that there was an overall increase in hindlimb perfusion in the combination group, as compared to the other groups including control. However, laser Doppler measurements are known to have great variability depending on the surrounding measuring conditions, site of measurement, or operator dependency [27, 28]. We tried to strictly adhere to a standardized protocol with all measurements being performed by one operator under a controlled environment, yet our measurements showed a wide range as a result of the intrinsic variability inherent to the laser Doppler perfusion measuring system. The lack of statistical significance in our perfusion measurements may probably be due to this variability, as shown by the large error bars in our graph.

Fibrosis and cell apoptosis were measured to assess the effects of angiogenesis at the histological level. The end result of ischemia is known to be cellular death and tissue remodeling by fibrotic scarring. Our results demonstrate that the combination group showed the lowest rate of cell death and fibrosis on TUNEL and Masson's trichrome stain, respectively, especially during the later phases (14 and 28 days). This demonstrates that the combination of MSCs with RADA increases angiogenesis, which leads to decreased cell death by improvement of ischemia and consequent lower tissue remodeling.

The use of SAPs to enhance the function of stem cells is very promising since the application of stem cells alone has major drawbacks in terms of survival and durability. Repeated injections and dosage modifications can overcome some of these drawbacks, but recent trends in treatment involve combinations of different treatment modalities to achieve the desired effects [29]. RADA in particular has been preferred for situations requiring angiogenesis due to its superiority in infiltrating endothelial and smooth muscle cells, inhibiting endothelial cell apoptosis and prolonging long-term survival [30, 31]. RADA provides a suitable 3-dimensional microenvironment where cells can adhere to and perform its normal cellular functions, which include endothelial and smooth muscle cell attachment, migration, and differentiation required for capillary-like structure formation [32]. The potential translational application of SAPs to clinical practice is also promising since these peptides are known to have the advantage of being nonimmunogenic and noninflammatory in nature [5, 33]. SAPs have been widely studied for the delivery of growth factors and drugs, but the use of stem cells is also very promising since the multipotency and self-renewing characteristics of stem cells allow for unlimited applications in the field of tissue engineering that cannot be easily achieved by single drugs or growth factors. However, studies involving the combination of stem cells and nanomaterials need to be performed in greater depth since it has been shown that phenotypic expression and function of stem cells are largely dependent on the microenvironmental cues in which the cells are exposed to, and therefore the fate of stem cells can vary depending on the type of nanomaterials used [34, 35].

Our study has several limitations, including the lack of statistical significance in some of the results despite showing a good tendency. The measurement of perfusion could have been improved in terms of variability by the use of laser Doppler imaging systems, which are known to be less sensitive to extrinsic factors. The effects of adding RSP to the combination group in improving angiogenesis also needs to be further delineated in future studies.

## 5. Conclusions

In this study, we demonstrated that MSCs combined with SAPs were able to enhance angiogenesis in ischemic hindlimbs of rats by promoting endothelial and smooth muscle cell proliferation, which led to a decrease in cell death and tissue remodeling in the form of fibrosis. In turn, the perfusion of the hindlimbs tended to improve as a result of increased angiogenesis. RADA also showed the ability to recruit endogenous MSCs into the site of action, especially so when a second SAP bound with substance P was added to allow for the dual presence of injected and intrinsically recruited MSCs at the site of action. Although preliminary in nature, the combination of MSCs and RADA can be an option for clinical application in ischemic disease conditions such as peripheral vascular diseases.

## Data Availability

Data is available upon request, please contact the corresponding author.
